# Repellency, toxicity, and anti-oviposition of essential oil of *Gardenia jasminoides* and its four major chemical components against whiteflies and mites

**DOI:** 10.1038/s41598-018-27366-5

**Published:** 2018-06-20

**Authors:** Tufail Ahmed Wagan, Wanlun Cai, Hongxia Hua

**Affiliations:** Hubei Insect Resources Utilisation and Sustainable Pest Management Key Laboratory, College of Plant Science and Technology, Huazhong Agricultural University, Wuhan, 430070 China

## Abstract

We examined the repellent, insecticidal, and anti-oviposition activities of the ethanol-extracted essential oil of *Gardenia jasminoides* against *Bemisia tabaci* and *Tetranychus urticae* adult and nymph stages under laboratory and greenhouse conditions. We used GC-MS to identify the main chemicals in the essential oil and selected four compounds, squalene, ethyl linoleate, n-hexadecanoic acid and 9–12-octadecadienoic, to evaluate their activity on whiteflies and mites. In the laboratory experiments, the essential oil of *G. jasminoides* showed maximum effects in fumigation against whitefly adults (81.48%) and contact toxicity against whitefly nymphs (46.44%), adult mites (49.81%), and mite nymphs (66.46%). In the greenhouse experiments, squalene showed maximum repellency against whitefly adults at 24 (89.59%) and 48 h (84.76%), whitefly nymphal toxicity at 24 (80.08%) and 48 h (77.28%), and mite toxicity at 24 (78.74%) and 48 h (73.86%). The essential oil showed maximum anti-oviposition activity against whiteflies at 24 (63.58%) and 48 h (59.56%) and toxicity to mite nymphs at 24 (82.45%) and 48 h (57.14%) in the bioassay. The essential oil had LC_50_ values of 2396.457, 2844.958, 56,990.975 and 21,468.619 against whitefly adults, whitefly nymphs, mite adults and mite nymphs, respectively. *G. jasminoides* and its major chemicals may effectively control the whiteflies and mites.

## Introduction

Numerous studies have confirmed that whiteflies (*Bemisia tabaci* [Gennadius, 1889]) and the two-spotted spider mite (*Tetranychus urticae* C.L. Koch, 1936) are pests with great economic impacts on many crops in greenhouses as well as in open fields worldwide^1^. *B. tabaci* is a small, 1–3 mm, sap-sucking pest from the family Aleyrodidae, order Hemiptera. Although whiteflies have a worldwide distribution, comprising more than 1,200 species, only a few species cause economic damage to horticulture and field crops^2,3^. Whiteflies feed on plant sap, causing stunted growth, especially in young plants, the silvering of leaves, the whitening of vegetable plants, and irregular colour and ripening processes in tomato and other fruits. Whiteflies secrete large amounts of honeydew during feeding, which may stimulate the growth of black mildew (sooty mould) and interfere with light absorption during photosynthesis in plants. Moreover, whiteflies transmit several kinds of plant viruses^4^. *T. urticae* are oval-shaped, approximately 0.4-mm-long pests from the family Tetranychidae, order Trombidiformes, that attack 1200 plant species in warm regions around the world^5–7^. The availability of host plants and favourable climates support rapid increases in its populations. The adults have a long life span and high birth rate, which cause maximum losses to host plants over a short time^8,9^. Adults persist on the underside of leaves and suck the sap throughout their lifetime. It has been estimated that each individual damages 18–22 plant cells per minute, causing white spot symptoms on leaves and premature leaf fall^10^. The individuals make a ball with their silk threads and are then dispersed by wind or attach to the surface of other organisms^11^.

Synthetic chemicals have been successfully used to manage insect species since the 1970s^1^. However, the excessive use of these chemical poisons has led to many environmental concerns, including negative effects on non-target species in natural agroecosystems, the disruption of the ecological equilibrium between pests and natural enemies, resistance of targeted species to pesticide, health hazards, and air pollution^12,13^. Using knowledge about the hazardous effects of pesticide application, scientists aim to develop new methods for effective pest control. For safe and effective control, many aromatic plant essential oils have been introduced for their repellency, contact and fumigant toxicity, regulation of growth, and pathogenic properties^14^. Essential oils have been used for a long time as insect repellents in stored products^15^. Not only are essential oils effective in pest management, but their chemical components are also effective against most insect species.

*Gardenia jasminoides* J. Ellis is an evergreen, flowering shrub from the family Rubiaceae. This species bears sweet, fragrant white flowers twice a year, in spring and summer^16^. It was introduced to western countries as an ornamental plant in the mid-18th century^17^. *Gardenia* and its chemical components have many medical and other beneficial properties, and the species has been grown in China for more than a thousand years owing to its fragrance and medical uses. Its essential oil is commonly used to treat infections, especially bladder injuries, jaundice, and abscesses, and to treat blood in the urine^18^. *Gardenia* spp. are toxic to some bacterial species, such as *Campylobacter jejuni*, with bactericidal activity (BA_50_) ranging from 0.003 to 0.009, and *Listeria monocytogenes*, with BA50 ranging from 0.057 to 0.092^19^.

To the best of our knowledge, the application of essential oil from *G. jasminoides* and its chemical components to repel, kill, or prevent oviposition in insects has remained unexplored. Our study aimed to identify the active chemical components of *G. jasminoides* essential oil through gas chromatography/mass spectrometry (GC-MS) analysis. Furthermore, it aimed to test the repellency, fumigant toxicity, and anti-oviposition activity of the essential oil and its components against whitefly adults and to test their contact toxicity against whitefly nymphs and mite adults and nymphs. The results will help in the development of methods to control these notorious pests in greenhouse environments.

## Results

### GC-MS analysis of the essential oil

*Gardenia jasminoides G. jasminoides* essential oil identified 14 major chemical components: isolongifolene, thujopsene, diethyl phthalate, 8.beta.h-cedran-8-ol, alpha-bisabolol, *n*-hexadecanoic acid, hexadecanoic acid, ethyl ester, ethylene brassylate, 9–12-octadecadienoic acid, ethyl linoleate, 9-octadecenoic acid ethyl ester, octadecanoic acid, ethyl ester, 9–12-octadecadienoyl chloride, and squalene. The percentages of these chemical components are shown in Table 1. On the basis of the percentages of the chemical components and previous studies, we selected four compounds, squalene, ethyl linoleate, n-hexadecanoic acid and 9–12-octadecadienoic acid, to evaluate their activity on whiteflies and mites.

**Table 1:** GC-MS results of the essential oils from *Gardenia jasminoides*.

Components	Retention Time	Percentage of Total	Formula
Isolongifolene	10.10	0.48	C15H24
Thujopsene	10.37	0.24	C15H24
Diethyl phthalate	11.21	0.14	C12H14O4
8.beta.h-cedran-8-ol	11.51	0.16	C15H26O
Alpha-bisabolol	11.81	0.10	C15H26O
n-Hexadecanoic acid	13.17	0.60	C16H32O2
Hexadecanoic acid, ethyl ester	13.30	0.76	C18H36O2
Ethylene brassylate	13.62	0.41	C15H26O4
9–12-Octadecadienoic acid	14.03	1.81	C18H32O2
Ethyl linoleate	14.12	1.30	C20H36O2
9-Octadecenoic acid ethyl ester	14.14	0.99	C20H38O2
Octadecanoic acid, ethyl ester	14.24	0.14	C20H40O2
9–12-Octadecadienoyl chloride	15.28	0.16	C18H31ClO
Squalene	17.54	0.37	C30H50

## Laboratory experiments

### Adult whitefly fumigation

**Figure 1:**
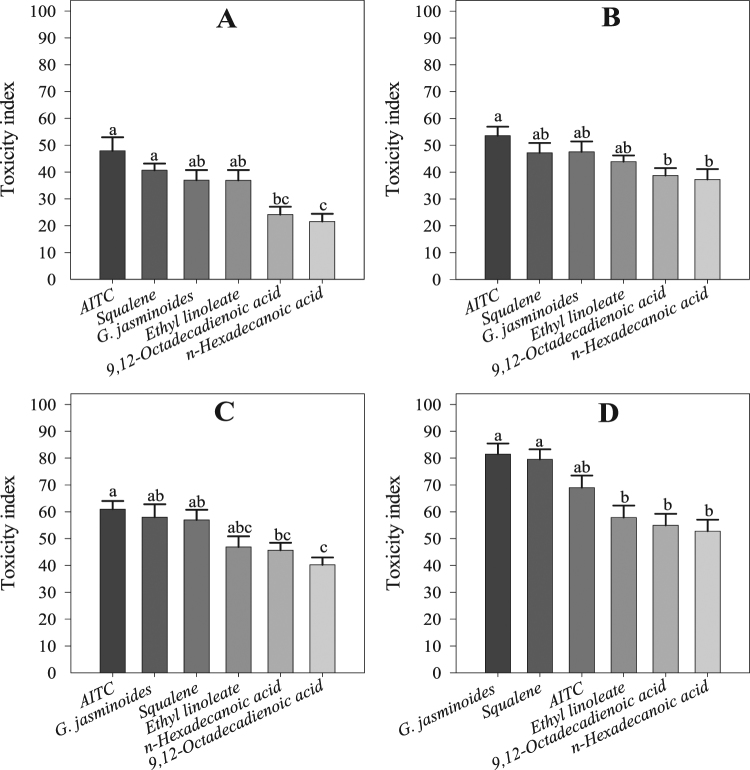
Fumigant toxicity to whitefly adults in the lab experiment. (**A**) Toxicity at 1 h, (**B**) toxicity at 3 h, (**C**) toxicity at 6 h, (**D**) toxicity at 9 h. Values are the means of 8 replicates. The mean numbers of adults were analysed using one-way ANOVA and Tukey HSD post hoc tests at a significance level of *P* < 0.05; means denoted by the same letter are not significantly different.

The essential oil from *G. jasminoides*, its four chemical compounds and Allyl isothiocyanate (AITC) displayed the highest fumigant toxicity against whitefly adults. Their toxicity increased with increasing time up to 9 h after the beginning of the bioassay. At the end, essential oil displayed the maximum toxicity (81.48 ± 3.98%), followed by squalene (79.48 ± 3.79%), AITC (68.99 ± 4.50%) ethyl linoleate (57.83 ± 4.48%), 9,12-octadecadienoic acid (54.98 ± 4.26%), and *n*-hexadecanoic acid (52.77 ± 4.25%) during the 9 h of the bioassay. The ANOVA showed significant differences among the components, including between the essential oil and the positive control (osthole) at 1 h (*F* = 7.81; df = 5; *P* < 0.00), 3 h (*F* = 3.29; df = 5; *P* < 0.01), 6 h (*F* = 5.36; df = 5; *P* < 0.01), and 9 h (*F* = 8.92; df = 5; *P* < 0.00) (Fig. 1).

### Whitefly nymph contact toxicity

**Figure 2:**
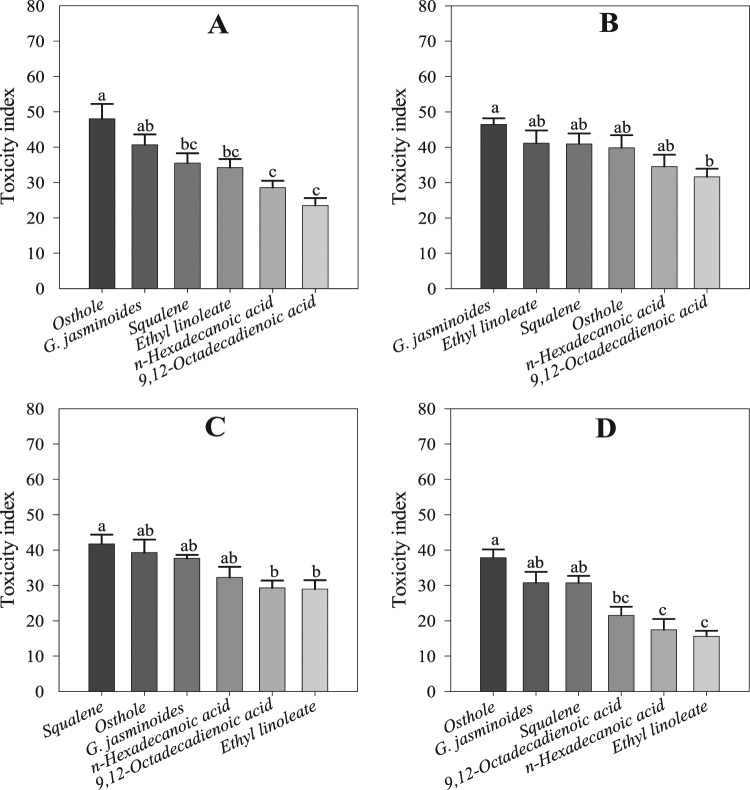
Contact toxicity to whitefly nymphs in the lab experiment. (**A**) Toxicity at 1 h, (**B**) toxicity at 3 h, (**C**) toxicity at 6 h, (**D**) toxicity at 9 h. Values are the means of 8 replicates. The mean numbers of adults were analysed using one-way ANOVA and Tukey HSD post hoc tests at a significance level of *P* < 0.05; means denoted by the same letter are not significantly different.

The essential oil, its components and osthole showed contact toxicity against the nymphal stage of the whitefly. The toxicity increased in the first 3 h of the bioassay but declined slowly thereafter. The highest contact toxicity (46.44 ± 1.76%) was observed for the essential oil, followed by that for ethyl linoleate (41.13 ± 3.61%), squalene (40.9 ± 2.99%), osthole (39.84 ± 3.57%), *n*-hexadecanoic acid (34.52 ± 3.39%), and 9,12-octadecadienoic acid (31.64 ± 2.29%) at 3 h after the beginning of the bioassay. The ANOVA revealed significant differences among all the treatments at 1 h (*F* = 9.26; df = 5; *P* < 0.00), 3 h (*F* = 3.25; df = 5; *P* < 0.01), 6 h (*F* = 3.83; df = 5; *P* < 0.01), and 9 h (*F* = 8.42; df = 5; *P* < 0.00) (Fig. 2).

### Adult mite contact toxicity

**Figure 3:**
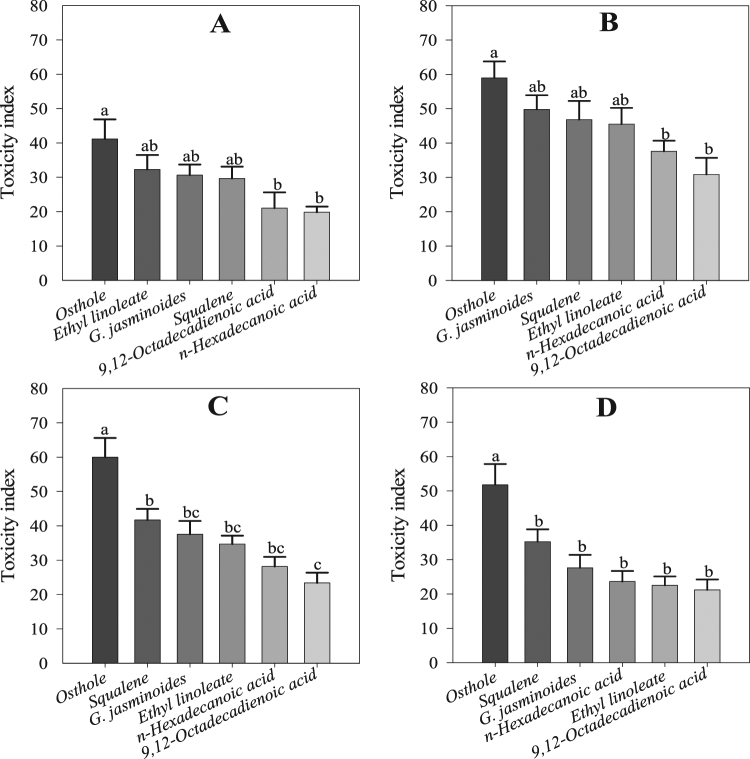
Contact toxicity to mite adults in the lab experiment. (**A**) Toxicity at 1 h, (**B**) toxicity at 3 h, (**C**) toxicity at 6 h, (**D**) toxicity at 9 h. Values are the means of 8 replicates. The mean numbers of adults were analysed using one-way ANOVA and Tukey HSD post hoc tests at a significance level of *P* < 0.05; means denoted by the same letter are not significantly different.

The contact toxicity of the essential oil from *G. jasminoides*, its four chemical components and osthole against adult mites was confirmed during the 9 h of the bioassay. The toxicity increased in the first 3 h of the bioassay, followed by a slight reduction afterward. Osthole showed the maximum toxicity (58.97 ± 4.80%), followed by the essential oil (49.81 ± 4.11%), squalene (46.81 ± 5.46%), ethyl linoleate (45.51 ± 4.74%), *n*-hexadecanoic acid (37.57 ± 3.12%), and 9,12-octadecadienoic acid (30.86 ± 4.83%). ANOVA revealed significant differences among the treatments, including between the essential oil and the four chemical components at 1 h (*F* = 3.87; df = 5; *P* < 0.01), 3 h (*F* = 4.57; df = 5; *P* < 0.01), 6 h (*F* = 12.32; df = 5; *P* < 0.00), and 9 h (*F* = 9.17; df = 5; *P* < 0.00) in the bioassay (Fig. 3).

### Nymph mite contact toxicity

**Figure 4:**
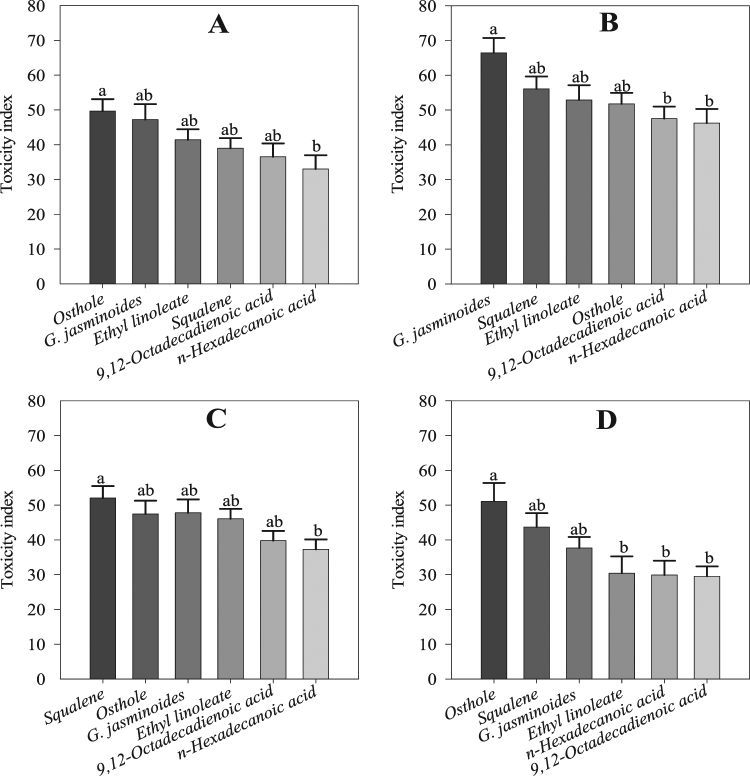
Contact toxicity to mite nymphs in the lab experiment. (**A**) Toxicity at 1 h, (**B**) toxicity at 3 h, (**C**) toxicity at 6 h, (**D**) toxicity at 9 h. Values are the means of 8 replicates. The mean numbers of adults were analysed using one-way ANOVA and Tukey HSD post hoc tests at a significance level of *P* < 0.05; means denoted by the same letter are not significantly different.

The essential oil, its chemical components and osthole showed greater toxicity against the nymphal stage of mites compared to that against adult mites; however, the toxicity did not last longer than 3 h, and it slowly declined later on. The highest toxicity was observed for *G. jasminoides* essential oil at 3 h in the bioassay (66.46 ± 4.26%), followed by squalene (56.11 ± 3.55%), ethyl linoleate (52.91 ± 4.22%), osthole (51.78 ± 51.78%), 9–12-octadecadienoic acid (40.83 ± 4.00%), and *n*-hexadecanoic acid (37.36 ± 3.26%). The ANOVA revealed significant differences among the treatments of the chemical components, *G. jasminoides* essential oil and osthole at 1 h (*F* = 3.04; df = 5; *P* < 0.02), 3 h (*F* = 3.66; df = 5; *P* < 0.01), 6 h (*F* = 2.80; df = 5; *P* < 0.03), and 9 h (*F* = 4.66; df = 5; *P* < 0.01) in the bioassay (Fig. 4).

## Greenhouse Experiments

### Repellency against adult whiteflies

**Figure 5:**
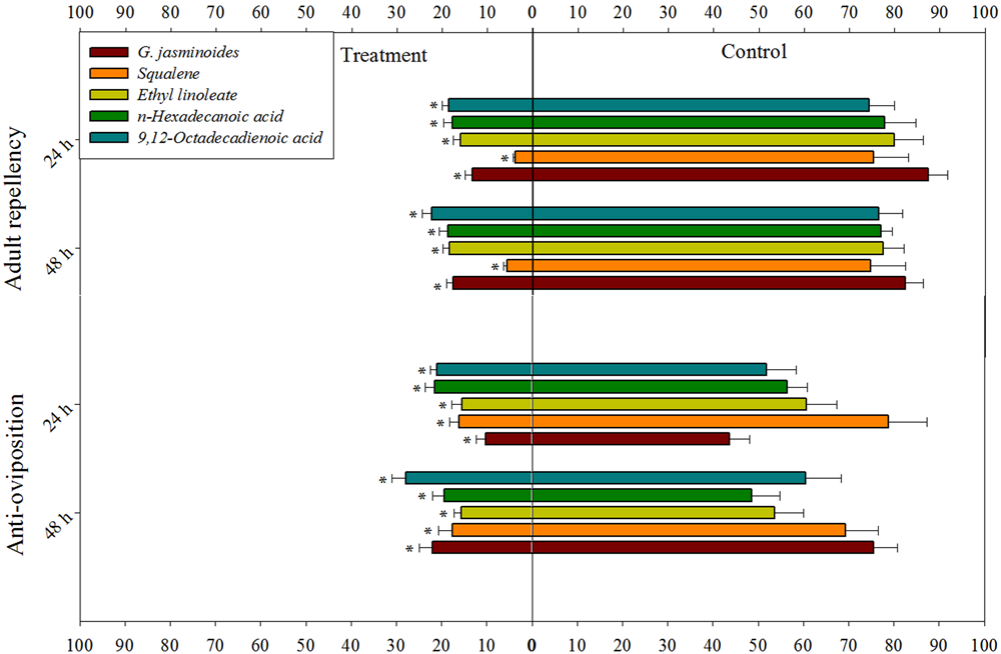
Whitefly repellency and oviposition in the greenhouse experiment at 24 and 48 h during the bioassay. Values are means of 8 replicates. The mean numbers of adults and eggs were compared using paired *t*-tests at a significance level of *P* ≤ 0.05. Asterisks indicate a significant difference between the control and treatment.

**Figure 6:**
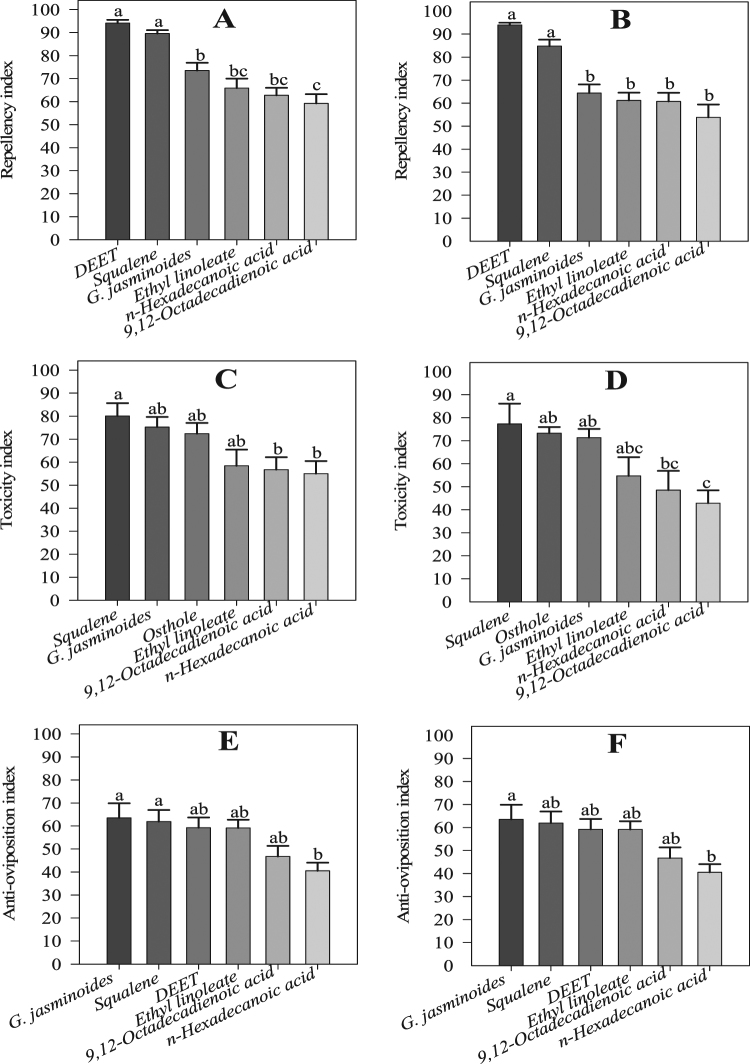
Whitefly repellency, nymph toxicity and oviposition in the greenhouse experiment: (**A**) repellency at 24 h, (**B**) repellency at 48 h, (**C**) toxicity at 24 h, (**D**) toxicity at 48 h, (**E**) oviposition at 24 h, (**F**) oviposition at 48 h during the bioassay. Values are means of 8 replicates. The mean numbers of adults, nymphs and eggs laid were analysed using one-way ANOVA and Tukey HSD post hoc tests (*P* < 0.05); means denoted by the same letter are not significantly different.

The results of the *t*-tests showed that *G. jasminoides* essential oil, its chemical components and the positive control (DEET) significantly repelled whitefly female adults in a greenhouse setting. Thus, there were significant differences in repellency between the controls and treatment with *G. jasminoides* essential oil (*t* = 15.06; df = 7; *P* < 0.00 and *t* = 12.16; df = 7; *P* < 0.00), squalene (*t* = 9.40; df = 7; *P* < 0.00 and *t* = 8.65; df = 7; *P* < 0.00), ethyl linoleate (*t* = 9.58; df = 7; *P* < 0.00 and *t* = 11.91; df = 7; *P* < 0.00), *n*-hexadecanoic acid (*t* = 9.76; df = 7; *P* < 0.00 and *t* = 15.01; df = 7; *P* < 0.00), 9–12-octadecadienoic acid (*t* = 9.07; df = 7; *P* < 0.00 and *t* = 7.97; df = 7; *P* < 0.00), and DEET (*t* = 26.90; df = 7; *P* < 0.00 and *t* = 42.02; df = 7; *P* < 0.00) at 24 and 48 h in the bioassay, respectively (Fig. 5). The ANOVA showed significant differences among the treatments of the application of the essential oil and its components at both 24 h (*F* = 21.70; df = 5; *P* < 0.00) and 48 h (*F* = 18.86; df = 5; *P* < 0.00). The greatest repellent activity was observed for DEET at 24 h, with 94.17 ± 1.39% of adult whiteflies being repelled, followed by squalene (89.59 ± 1.43%), *G. jasminoides* essential oil (73.51 ± 3.34%), ethyl linoleate (65.91 ± 4.07%), *n*-hexadecanoic acid (62.78 ± 3.26%), and 9–12-octadecadienoic acid (59.24 ± 4.02). However, the repellency percentage slightly decreased over time (Fig. 6).

### Contact toxicity to whitefly nymphs

The ANOVA showed significant toxicity of all components, osthole, including the essential oil, against the nymphs at 24 h (*F* = 3.89; df = 5; *P* < 0.01) and 48 h (*F* = 4.74; df = 5; *P* < 0.01) in the bioassay. The maximum toxicity was recorded at 24 h for squalene (80.08 ± 5.56%), followed by *G. jasminoides* essential oil (75.25 ± 4.39%), osthole (72.40 ± 4.69%), ethyl linoleate (58.46 ± 7.00%), 9–12-octadecadienoic acid (56.73 ± 5.47%), and *n*-hexadecanoic acid (55.03 ± 5.49%); at 48 h, the maximum toxicity slightly increased for squalene (77.28 ± 8.80%) and osthole (73.31 ± 2.62%) but decreased for *G. jasminoides* essential oil (71.30 ± 3.82%), ethyl linoleate (54.69 ± 8.10%), *n*-hexadecanoic acid (48.56 ± 8.30%), and 9–12-octadecadienoic acid (42.85 ± 5.58%) (Fig. 6).

### Oviposition deterrence of whiteflies

The *t*-test results of the choice experiment showed that all components, including the essential oil, have strong oviposition deterrence effects on whitefly females. There were significant differences between the controls and *G. jasminoides* essential oil (*t* = 6.44; df = 7; *P* < 0.00 and *t* = 7.15; df = 7; *P* < 0.00), squalene (*t* = 8.16; df = 7; *P* < 0.00 and *t* = 10.25; df = 7; *P* < 0.00), ethyl linoleate (*t* = 8.22; df = 7; *P* < 0.00 and *t* = 7.18; df = 7; *P* < 0.00), *n*-hexadecanoic acid (*t* = 6.81; df = 7; *P* < 0.00 and *t* = 4.29; df = 7; *P* < 0.00), 9–12-octadecadienoic acid (*t* = 5.59; df = 7; *P* < 0.00 and *t* = 5.40; df = 7; *P* < 0.00), and DEET (*t* = 10.01; df = 7; *P* < 0.00 and *t* = 15.85; df = 7; *P* < 0.00) at 24 and 48 h in the bioassay, respectively (Fig. 5). The results of the ANOVA revealed significant differences among the treatments, including the essential oil and its components and DEET, at 24 h (F = 4.24; df = 5; *P* < 0.01) and 48 h (*F* = 3.52; df = 5; *P* < 0.01). The greatest oviposition deterrence activity was observed for *G. jasminoides* essential oil (63.58 ± 6.29%) at 24 h in the bioassay, followed by squalene (61.95 ± 5.02%), DEET (59.23 ± 4.47%), ethyl linoleate (59.14 ± 3.60%), 9–12-octadecadienoic acid (46.74 ± 4.62%), and *n*-hexadecanoic acid (40.52 ± 3.54%). At 48 h, the oviposition deterrence activity of *G. jasminoides* essential oil (59.56 ± 5.84%), squalene (55.73 ± 4.74%), DEET (55.44 ± 2.96%), ethyl linoleate (54.02 ± 3.02%), *n*-hexadecanoic acid (40.84 ± 8.09%), and 9–12-octadecadienoic acid (35.51 ± 4.38%) was slightly reduced (Fig. 6).

### Contact toxicity to adult mites

**Figure 7:**
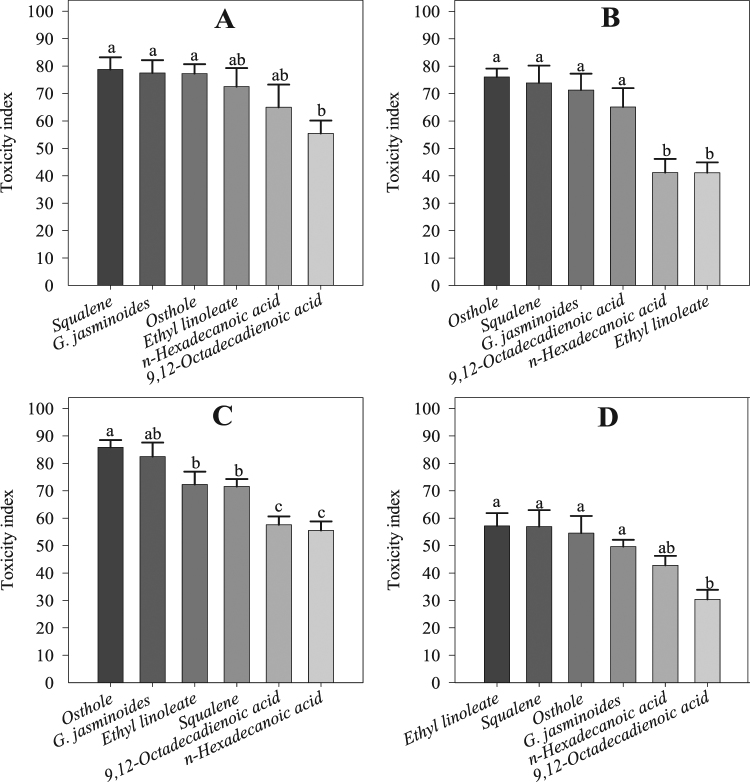
Mite adult and nymph toxicity in the greenhouse experiment: (**A**) Toxicity to adults at 24 h, (**B**) toxicity to adults at 48 h, (**C**) toxicity to nymphs at 24 h, (**D**) toxicity to nymphs at 48 h during the bioassay. Values are means of 8 replicates. The mean numbers of adults and nymphs were analysed using one-way ANOVA and Tukey HSD post hoc tests (*P* < 0.05); means denoted by the same letter are not significantly different.

All the tested chemicals and the essential oil caused mortality in adult mites. The ANOVA showed significant differences among the treatments at 24 h (*F* = 3.58; df = 5; *P* < 0.01) and 48 h (*F* = 12.12; df = 5; *P* < 0.00) in the bioassay. The maximum toxicity of 78.74 ± 4.44% was observed for squalene at 24 h, followed by *G. jasminoides* essential oil (77.47 ± 4.65%), osthole (77.26 ± 3.40%), ethyl linoleate (72.50 ± 6.76%), *n*-hexadecanoic acid (64.96 ± 8.26%), and 9,12-octadecadienoic acid (55.43 ± 4.68%). At 48 h, the toxicity was slightly reduced for each treatment, including the positive control. Osthole showed the maximum toxicity (76.01 ± 3.11%), followed by squalene (73.86 ± 6.33%), *G. jasminoides* essential oil (71.26 ± 5.96%), 9,12-octadecadienoic acid (65.06 ± 6.90%), *n*-hexadecanoic acid (41.20 ± 4.92%), and ethyl linoleate (41.13 ± 3.77%) (Fig. 7).

### Contact toxicity to mite nymphs

The results of the ANOVA indicated a significant difference between the four compounds, the essential oil and the positive control at 24 h (*F* = 15.01; df = 5; *P* < 0.00) and 48 h (*F* = 7.92; df = 5; *P* < 0.00) in the bioassay. All four compounds, the essential oil, and osthole were toxic to the mite at the nymphal stage, but at 24 h, the toxicity of osthole (85.83 ± 2.64%), the essential oil (82.45 ± 5.10%), ethyl linoleate (72.28 ± 4.67%), squalene (71.52 ± 2.77%), 9–12-octadecadienoic acid (57.61 ± 3.02%), and *n*-hexadecanoic acid (55.54 ± 3.26%) was higher compared to the toxicity (57.14 ± 4.70%, 56.89 ± 6.05%, 54.52 ± 6.30, 49.65 ± 2.43%, 42.77 ± 3.48%, and 30.37 ± 3.49%, respectively) at 48 h (Fig. 7).

### Toxic effects of G. jasminoides essential oil on whiteflies and mites

The toxicity of the essential oil against whiteflies is presented in Table 2. The essential oil of *G. jasminoides* showed toxicity to both the mature and immature stages of whiteflies. An LC_50_ for whiteflies in the adult stage was recorded at 2396.457 ppm, followed by an LC_30_ of 1154.348 ppm, and an LC_10_ of 402.072 ppm, while an LC_50_ of 2844.958 ppm, an LC_30_ of 1428.650 ppm, and an LC_10_ of 528.446 ppm were recorded for whitefly nymphs. Toxicity of the essential oil was also observed for mites in the nymph and adult stages. We recorded an LC_50_ of 56,990.975 ppm, an LC_30_ of 9171.939 ppm, and an LC_10_ of 656.141 ppm for mites in the adult stage. Similarly, an LC_50_ of 21,468.619 ppm, an LC_20_ of 4443.403 ppm, and an LC_10_ of 457.089 ppm were recorded for mite nymphs (Table 3).

**Table 2:** Toxicity of *G. jasminoides* essential oil against nymph and adult whiteflies.

EO	Insects	Essential oil (10,000 ppm concentration)					
		N^a^	LC_10_	LC_30_	LC_50_	Slope ± SE	χ^2^ (df)
*G. jasminoides*	Nymph	3054	528.446 (289.867–88.745)	1428.650 (998.956–1860.801)	2844.958 (2225.040–3566.546)	1.753 ± 0.064	20.018 (4)
*G. jasminoides*	Adult	960	402.072 (81.305–819.789)	1154.348 (461.561–1871.500)	2396.457 (1376.598–3699.940)	1.653 ± 0.11	20.197 (4)

EO; essential oil of *G. jasminoides*.

^a^Number of insects/subjects.

χ^2^ is statistically significant (P < 0.05).

**Table 3:** Toxicity of *G. jasminoides* essential oil against nymph and adult mites.

EO	Insect	Essential oil (10,000 ppm concentration)					
		N^a^	LC_10_	LC_30_	LC_50_	Slope ± SE	χ^2^ (df)
*G. jasminoides*	Nymph	1219	457.089 (198.633–741.177)	4443.403 (3389.503–6304.383)	21468.619 (12879.293–52137.015)	0.767 ± 0.115	1.060 (3)
*G. jasminoides*	Adult	1216	656.141 (251.016–1082.636)	9171.939 (6118.797–18870.074)	56990.975 (25215.473–302757.02)	0.661 ± 0.121	2.312 (3)

EO; essential oil of *G. jasminoides*.

^a^Number of insects/subjects.

χ^2^ is statistically significant (P < 0.05).

## Discussion

The essential oil extracted from *G. jasminoides*, its four major components, and the positive control showed maximum fumigant toxicity against whitefly adults in airtight glass containers and toxicity to the nymphal stage; *G. jasminoides* essential oil was the most toxic compared to its chemical components. Similar results of fumigant toxicity were reported by Wagan *et al*.^20^ for the ethanol-extracted essential oil of *Acorus tatarinowii*, whose fumigant toxicity to whitefly adults was 98.8% after 8 h during a bioassay. Extracts of *G. jasminoides* fruits (at 10,000 ppm) have lethal effects against *M persicae* and *A. gossypii*^21^. The essential oil from *G. jasminoides* has traditionally been used to treat irritability, urinary tract infections, headaches, and tension for 2,000 years^18^. Its chemical components have been identified in other plants and studied in the clinical treatments of many disorders. For example, the bark and leaves of *P. alatum* contain squalene, *n*-hexadecanoic acid, and 9,12-octadecadienoic acid, which show anti-inflammatory, anti-tumour, anti-cancer, anti-oxidant, anti-microbial, and immunostimulant activity^22^. However, ethyl linoleate is toxic to aquatic organisms^23^. In our study, toxicity increased in the first 3 h of the bioassay, and then it slightly decreased, indicating that the treatments exhibited continuous toxic activity against whitefly adult and nymphal stages.

*G. jasminoides* essential oil showed the greatest toxicity against mite adult and nymphal stages compared to the toxicity of its individual chemical components; the toxicity lasted for 9 h. Toxic properties of *Gardenia* spp. essential oil were also reported by Friedman *et al*.^19^ against bacterial species. Moreover, Kim *et al*.^21^ observed significantly lower reproductive activity in *T. urticae* when exposed to extracts of *G. jasminoides* peels and fruits at 10,000 ppm for up to three days. Smith *et al*.^24^ showed that some plants. such as *Azadirachta indica*, contain squalene, which inhibits the feeding of larval and adult insects. *n*-Hexadecanoic acid is found in different tissues in plants and animals, and its role varies in different applications^25^. Bergsson *et al*.^26^ reported that 9,12-octadecadienoic acid (linoleic acid) is lethal to *Candida albicans*. In the present study, squalene toxicity against nymphs was greater than that against adults, indicating that adults are less sensitive to the effects of squalene and can resist some toxicity compared to the nymphs.

Treatment with *G. jasminoides* essential oil, squalene, ethyl linoleate, *n*-hexadecanoic acid, and 9–12-octadecadienoic acid protected the plants and kept adult whiteflies away from the treated plants for up to 48 h, when these compounds exhibited the highest mortality. The treatments also disrupted the laying of eggs by adults. Methanol extracts of *G. jasminoides* peels and fruits at 10,000 ppm showed slight repellent activities against *T. urticae* at 24 h after treatment. However, this repellency activity decreased at 72 h^21^. Similar results in terms of repellency, toxicity, and anti-oviposition activity against *Bemisia tabaci* were reported by Yang *et al*.^27^ for the essential oil of *Pogostemon cablin*, which showed the highest repellency against adult *B. tabaci*, for the essential oil of *Thymus vulgaris*, which showed the greatest contact toxicity to nymphs, and for the essential oil of *Corymbia citriodora*, which reduced the number of eggs laid on treated plants. Furthermore, Yadav and Mendhulkar^28^ also recorded adult repellency, nymphal mortality, and egg-deterrent activities for the essential oil extracted from *Couroupita guianensis*. The greatest repellency, 66.11% and 62.46%, against greenhouse whiteflies was observed for the essential oils of *Cuminum cyminum* 3 days after the start of a bioassay and *Thymus vulgaris* at 6 days, respectively, whereas the essential oil from *Achillea millefolium* reduced oviposition by 67.77% by day 3^29^. In our study, squalene caused the greatest adult repellency and nymph mortality. However, squalene naturally occurs in and is a vital part of all plants and animals, but it exhibits acute toxicity whenever it is used in cosmetics^30^. The lowest number of eggs was deposited on plants treated with *G. jasminoides* essential oil, indicating that either the essential oil or its individual chemical components can be used as oviposition deterrents. Furthermore, *Feronia limonia* leaves contain *n*-hexadecanoic acid, which has been found to be toxic to the fourth larval stage of *Aedes aegypti*, *Culex quinquefasciatus*, and *Anopheles stephensi*, with LC_50_ values of 57.23, 129.24, and 79.58 ppm, respectively^31^. The Environmental Protection Agency (EPA) has reported that 9,12-octadecadienoic acid (linoleic acid) has anti-microbial activity against some species, such as *Listeria monocytogenes, Bacillus subtilis, Pseudomonas aeruginosa*, and *Staphylococcus aureus*^32^. Taken together, the results presented here and those reported in other studies indicate that the essential oil of *G. jasminoides* and its major chemical components exhibit repellent, toxic, and anti-oviposition activity against *B. tabaci*.

The essential oil and its main chemical components caused the mortality of adult and nymphal mites and thus controlled this pest. Toxicity of essential oils against mites has also been described in other studies. For example, Motazedian *et al*.^33^ compared the toxic effects of essential oil extracted from *Mentha longifolia* (Lamiaceae) against *Tetranychus urticae* (LC_50_ value of 20.08 µL L^−1^ air) with that of *Salvia officinalis* (Lamiaceae) and *Myrtus communis* (Myrtaceae) (LC_50_ value 53.22, 60.93 µL L^−1^ air). In our study, squalene expressed the highest toxicity and anti-feeding effects against adult mites. Plants such as *Azadirachta indica* contain squalene, which inhibits the feeding of insects at young stages^24^. Our results corroborate those of Parthipan *et al*.^22^ who reported that the leaves as well as the bark of *P. alatum*, due to the presence of squalene, 9,12-octadecadienoic acid, and *n*-hexadecanoic acid, exhibit anti-microbial, anti-inflammatory, anti-oxidant, anti-cancer, anti-tumour, and immunostimulant activities. Essential oil from *G. jasminoides* showed the maximum lethal activity against mite nymphs in the first 24 h of the assay; its toxicity was reduced afterwards, while the toxicity of ethyl linoleate peaked. A recent study on the effect of *Gardenia* yellow powders with geniposide on rats, in which the rats were fed 60 mg kg^−1^ day^−1^ for 3 months, did not detect any significant toxic effect of this compound on the liver, kidneys, or other organs^34^. We can conclude that *Gardenia* has a low toxic effect on insects, whereas its essential oil hinders the feeding of insects. Moreover, the mortality rate of adult mites was constant over the first 48 h of the experiment in the greenhouse, whereas the toxicity to nymphs lasted for a lower duration compared to that in adults. These results suggest that the existing nymphs died suddenly after the bioassay, but new nymphs emerged from eggs. Therefore, the observed fluctuations in the results indicated low toxic effects of the compounds and essential oil on the nymphal stage.

Our results showed insecticidal and acaricidal effects of *Gardenia jasminoides* essential oil, with LC_50_ values of 2396.46 ppm, 2844.958 ppm, 56,990.975 ppm and 21,468.619 ppm against whitefly adults, whitefly nymphs, mite adults and mite nymphs, respectively. There is a lack of studies on the insecticidal activity of essential oils, particularly *Gardenia jasminoides* essential oil. However, many studies have described that essential oils have repellent properties against insect and mite pests under laboratory, greenhouse and field conditions.

In conclusion, *G. jasminoides* essential oil and its major chemical components may effectively control the adult and nymphal stages of whiteflies and mites and affect oviposition in whiteflies. The repellency, anti-oviposition, fumigation and toxicity of this essential oil and its four chemical components were similar to those of positive controls. This is the first study on the effects of *G. jasminoides* essential oil and its four chemical components against whitefly and mite pests. Further research should examine the effectiveness of this essential oil on other economic pests in greenhouse and open field settings.

## Methods

### Host plants and insect culture

Seeds of the susceptible tomato variety ‘Xian zao hong’ were sown in plastic pots (13 cm high × 15 cm wide, filled with 1.5 kg of equal proportions of soil and organic material). After 15 days of germination, single tomato plants were transplanted into pots, and 25 days after transplanting, when the plants had developed 35–40 leaves, they were used in the greenhouse experiments. Whiteflies and mites were reared on tomato plants for more than 2 years without any pesticide application in a greenhouse at 60 ± 10% relative humidity and 25 ± 5 °C. The experiment was conducted from July to December 2016 at the Hubei Insect Resources Utilization and Sustainable Pest Management Key Laboratory, Huazhong Agricultural University, Wuhan, China.

### Ethanol extraction of G. jasminoides

Extraction of plant material was based on the method described by Su *et al*.^35^ with some modifications. Fruits of *G. jasminoides* were purchased from a plant shop in Wuhan, a franchise of the Beijing Tongrentang Group, China. The seeds were cleaned and oven dried for 3–5 days at 45 °C until they reached a constant weight. The dried material was then crushed in a shredder (De Qing Baijei, China) and passed through a sieve with 40-mm mesh. The plant powder (1 g) was extracted in 5 mL of 95% ethanol in the dark at room temperature (20–25 °C) for 7 days; the bottles with the plant powder were shaken twice a day for maximum mixing and to dissolve the powder in the ethanol. The solvent was filtered, and the residuals were again dissolved using the same solution with a ratio of 2.5 mL of ethanol to 1 g of residuals under the same conditions. Both filtered solutions (first and second filtration) were mixed and dried in a rotary evaporator until all the liquid evaporated. The final extract had a glue-like consistency with an oil-type liquid on the surface. The extract was weighed; a total of 159.28 g of extract was obtained from 589.54 g of seeds. The extract was transferred into a brown collection bottle and stored at 4 °C. The obtained crude extract of *G. jasminoides* (0.05 g) and 0.05 mL of chemical components and DEET were dissolved separately in 0.3 mL of dimethyl sulfoxide (DMSO); 1% Tween 20 was added to the solution, and the final volume was adjusted to 5 mL at a final concentration of 10,000 ppm by adding double distilled water. Osthole and Allyl isothiocyanate were dissolved in the same amount of water as the other solutions. These solutions were then used in separate treatments as a test solution.

### Laboratory experiment

#### Fumigant toxicity against whitefly adults

The test solution (1 mL) was pipetted onto a filter paper that was 6 cm in diameter, and the same volume of a solution containing Tween 20 and DMSO was applied to another filter paper and used as a control. When the liquids dried, individual paper discs were attached to the lids of 100-mL glass jars containing 20 five-day-old whitefly adults, and the lids were sealed with polyethylene strips to avoid any ventilation. The jars were maintained at 25 ± 2 °C, 50 ± 5% relative humidity and a photoperiod of 14 h light:10 h dark. Insect mortality was recorded at 1, 3, 6, and 9 h from the beginning of the bioassay. Each treatment was replicated eight times.

#### Contact toxicity against adult and nymph mites and whitefly nymphs

Two proximate leaves on the stem of a tomato plant hosting mites were selected for this assay. The test solution (0.1 mL) was carefully applied to the leaves using a cotton swab; the same volume of the control solution was applied to the control leaves. The leaves were placed in cages, and the stem was placed in water in a vessel held under the cage. The mortality was recorded at 1, 3, 6, and 9 h from the beginning of the bioassay. The experiment was repeated with leaves hosting mite nymphs and whitefly nymphs. Each treatment was replicated eight times.

### Greenhouse experiment

#### Whitefly adult repellency and anti-oviposition effects

Tomato plants with 35–40 fully expanded leaves were sprayed with 10 mL of the prepared test solution using a plastic hand sprayer, and the same volume of a solution containing only DMSO and Tween 20 was sprayed onto the plants used as controls. A total of 10 treated and 10 control plants were transferred into a glasshouse (3.30 m high, 6.50 m long, 3 m wide) 30 min after the spraying. Approximately 1000 five-day-old whitefly female adults were released into the glasshouse containing the plants. The plants were checked early in the morning, when whiteflies are passive, 24 and 48 h after application. During each recording, 50 leaves were randomly collected from each treatment and control, and the oviposited eggs were examined under a stereo microscope. There were eight replicates for each treatment.

#### Toxicity against adult and nymph mites and whitefly nymphs

The plants infested with mites and whitefly nymphs were sprayed with a hand sprayer using 10 mL of the test solution and used as a treatment, and the plants sprayed with the control solution were used as a control. The potted plants were kept in the glasshouse under the same environmental conditions as those used in the experiment with whitefly adults. After 24 and 48 h, 10 leaves were randomly collected from each plant and observed to calculate the percent mortality using the formula Mortality = Total number of insects – Number of live insects.

#### Identification of chemical components using GC-MS

The identification of the chemical components of the essential oil was carried out using GC-MS with a Varian 450-GC/320-MS (Agilent Technologies, Inc., Santa Clara, CA, USA). An HP-5 MS capillary column (the thickness of the film was 30 m × 0.25 mm i.d. × 0.25 μm) was used with a flame ionization detector. For the GC, the starting injector oven temperature was maintained at 60 °C for 3 min, ramped at 10 °C/min to 180 °C for 1 min, and then ramped at 20 °C/min to 280 °C for 15 min. The samples (1 μL), diluted to 1% with hexane, were injected at a split ratio of 1:10 and column pressure of 100 KPa. Helium gas was used at the rate of 1.0 mL/min as the sample carrier. The MS quad, ion source, and transmission line temperatures were set to 150 °C, 230 °C, and 250 °C, respectively. The chemical constituents were identified from the gas chromatography using the online libraries of Wiley, REPLIB, MANLIB, and PMWTox3N. Based on the GC-MS results and previous research on bioactivity^22–24,30^, four major chemical components, squalene, ethyl linoleate, 9,12-octadecadienoic acid, and n-hexadecanoic acid, were selected for their bioactivity against whiteflies and mites and purchased from Tokyo Chemical Industry Co., Ltd., Japan.

### Statistical analysis

The percent repellency (PR) was calculated according to Liu *et al*.^36^: PR (%) = [(C − T)/(C + T)] × 100. *T*-tests were used to compare insect repellency and oviposition between the treatments and the control. Analysis of the LC_50_ was performed with PoloPlus. One-way analysis of variance (ANOVA) was used to compare the differences between the means of the treatments with the Tukey’s honest significant difference (HSD) test; *P* < 0.05 was considered significant. For the data analysis, we used SPSS version 20 (IBM Corp., Armonk, NY, USA). The percentage data were arcsine–square root transformed, and all count data were square root (x + 1) or log 10 (x + 1) transformed before being subjected to data analysis. The untransformed means are presented in the results. The figures were created using SigmaPlot 10.0 (Systat Software, Inc., San Jose, CA, USA).
